# When Overweight Is the Normal Weight: An Examination of Obesity Using a Social Media Internet Database

**DOI:** 10.1371/journal.pone.0073479

**Published:** 2013-09-18

**Authors:** Meghan Kuebler, Elad Yom-Tov, Dan Pelleg, Rebecca M. Puhl, Peter Muennig

**Affiliations:** 1 Department of Health Policy and Management, Mailman School of Public Health, Columbia University, New York, New York, United States of America; 2 Microsoft Research Israel, Herzeliya, Israel; 3 Yahoo! Labs, Haifa, Matam Park, Israel; 4 Rudd Center for Food Policy and Obesity, Yale University, New Haven, Connecticut, United States of America; University of Catania, Italy

## Abstract

Using a large social media database, Yahoo Answers, we explored postings to an online forum in which posters asked whether their height and weight qualify themselves as “skinny,” “thin,” “fat,” or “obese” over time and across forum topics. We used these data to better understand whether a higher-than-average body mass index (BMI) in one’s county might, in some ways, be protective for one’s mental and physical health. For instance, we explored whether higher proportions of obese people in one’s county predicts lower levels of bullying or “am I fat?” questions from those with a normal BMI relative to his/her actual BMI. Most women asking whether they were themselves fat/obese were not actually fat/obese. Both men and women who were actually overweight/obese were significantly more likely in the future to ask for advice about bullying than thinner individuals. Moreover, as mean county-level BMI increased, bullying decreased and then increased again (in a U-shape curve). Regardless of where they lived, posters who asked “am I fat?” who had a BMI in the healthy range were more likely than other posters to subsequently post on health problems, but the proportions of such posters also declined greatly as county-level BMI increased. Our findings suggest that obese people residing in counties with higher levels of BMI may have better physical and mental health than obese people living in counties with lower levels of BMI by some measures, but these improvements are modest.

## Introduction

Obesity poses a significant threat to population health [1]. Over 7 million quality-adjusted life years are lost annually as a result of excess body weight in the U.S. alone [2]. One projection suggests that the rapid rise in obesity will lead to the first measured decline in life expectancy in the U.S. since the turn of the 20th century [3]. However, recent findings suggest that a subgroup of obese persons do not have metabolic or cardiovascular abnormalities typically linked to obesity, and in fact, this subgroup of approximately 10–25% of obese persons, may even be at lower risk for cardiovascular disease than normal weight persons with metabolic risk factors [4], [5]. There is also evidence that psychological factors, such as stigmatization, might play a role not only in mental health but also in the physical health of obese persons, possibly inducing the metabolic changes associated with obesity [6], [7]. If stigmatization plays a role in determining the health of overweight or obese people, then overweight or obese people who live in counties with higher than average body mass index ([BMI] measured as kg/m^2^) may experience better health than those who live in counties with lower than average BMIs because obesity is more normative. In this paper, we examined whether people who live in counties with higher than average BMIs experience obesity in different ways than those who are in counties with average or lower than average BMIs.

To examine this question, we used a social media database, which offers a large sample size, is prospective in that future postings on health by the same respondent can be recorded, and can provide unique insight into people’s actual perceptions or insecurities. To date, knowledge about how people think, feel, and respond to their changing bodies has mostly come from formal surveys with questions defined by research scientists and directed at volunteer respondents [8]–[10]. By instead investigating content provided during natural conversations, it is possible to overcome social desirability bias associated with stigmatized topics [11], [12], and uncover previously unexplored questions that are a priority to the public but not necessarily easily studied by researchers. For example, it becomes possible to observe a broader range of potentially “unhealthy” weight related concerns among the public such as bullying, the mental and physical effects of a distorted body image, and obesity itself without actually asking the respondent stigmatizing questions.

Social media represent a form of electronic communication through which users create online (internet) communities to share information, ideas, personal messages, and other content [13]. The internet allows for the collection of data with unmatched breadth, depth, and scale [14]–[16]. By 2008, 75% of internet users used social media, an increase of nearly 20% from the year prior. This equates to hundreds of millions of active users [17]. While such samples are by no means representative of the general population, they can nevertheless provide “real world” observations of the general public’s perceptions of their own body size, opening the door to new types of scientific studies.

For instance, one factor that is perhaps best examined using social media and is often ignored in scientific and policy-related discourse about overweight/obesity is the pervasive stigmatization of overweight/obese individuals. Rates of weight discrimination are comparable to racial discrimination [18], and appear to be increasing over time [19]. Overweight/obese persons face stigmatization in multiple settings, including the workplace, health care institutions, school, and at home [20], [21]. Among adolescents, being overweight/obese is a common pretense for teasing and bullying at school [22], though this can also happen to underweight persons as well [23]. In areas where obesity is more common, obese persons may face less stigmatization and incur fewer undesirable social outcomes of obesity, such as bullying, simply because it is more normative. As a result, one might expect those of a given BMI in counties with many obese or overweight people to have better physical and mental health outcomes than those of a similar BMI in counties with fewer obese or overweight people.

In addition, self-perceived weight status may vary from actual weight [9] and some individuals may perceive themselves to be overweight when their weight is actually in a healthy weight range [24]. Regardless of whether one actually is overweight, feeling overweight/obese may serve as a source of chronic psychological stress [25], [26]. Chronic psychological stress, in turn, can lead to autonomic dysregulation, a risk factor for diabetes, hypertension, hypercholesterolemia, and heart disease [27]–[29]. Those who are at a healthy weight but see themselves as “fat” or “obese” may serve as a useful counterfactual for measuring body-image-associated psychological stress relative to those who are at a healthy weight and accurately see themselves this way [7], [18], [25], [26]. We therefore examined whether such health concerns arise among healthy weight persons who ask whether they are “fat” or “obese” as a measure of one’s view of his or her body image. This is important because medical conditions associated with the stress response overlap with the medical conditions associated with overweight/obesity [28]. That is, both stress and overweight/obesity are thought play a central role in glucose metabolism (both in production and cellular uptake via insulin), blood pressure regulation, and lipid regulation [30].

One’s self-perceived weight is related to how one’s weight compares to those around them. For instance, in an area of the Dominican Republic where obesity is not stigmatized and overweight bodies are desirable, researchers found satisfaction with one’s weight among overweight participants who compared their weight to the norm or desired norm of those around them [27].

In the present study, we focused on participants who posted their height and weight, allowing us to calculate the poster’s BMI. Using the poster’s county of residence, we explore whether a higher mean BMI within the poster’s county predicts the types of questions that they ask about their body or the other types of health questions that the poster might go on to post at a later date. Specifically, we explored responses both among those who are at a healthy weight (based on calculated BMI) but nevertheless asked whether they are “fat” or “obese” and those who asked these questions and did in fact meet BMI definitions for overweight/obesity. We then reviewed future postings of public health importance (e.g., bullying) or general health concerns possibly related to obesity (e.g., heart disease, diabetes, and fertility issues [3], [31]). We examined responses both within the context of county mean BMI levels and the poster’s actual BMI level. Our aim was not to establish causal links, but rather to use a unique dataset to identify trends within social media postings related to body weight perceptions.

## Materials and Methods

Yahoo Answers (http://answers.yahoo.com) is a web site that allows askers to anonymously post questions online and receive answers from other anonymous askers. For the present study, we analyzed individuals who posted questions about body weight (“askers”). When posting a question, the user categorizes it into one of approximately 1,700 categories, including several weight related categories, which are arranged in a hierarchy. It is possible to study such data while maintaining a posters’ anonymity by internally linking all of a registered users’ posts, but de-linking this information to the users’ identifying information. This creates a de-identified database that employees of Yahoo can use to analyze online posts.

Since its inception in 2005, more than 200 million questions and over one billion answers have been posted to Yahoo Answers. Askers are located around the world, but are predominantly from the United States. The web site is limited to askers aged 13 or older. We obtained Yahoo privacy and human subject research approval for the use of the data, and all data were anonymous and observational (i.e., previously posted questions were analyzed, no users were approached by the researchers, and identifying information was not released). The text of the questions and the answers is publicly available. Other data (demographics, etc.) may be available to researchers following a signing of the necessary legal agreements to maintain user privacy. The analysis reported in this paper was performed using Matlab version 8.1 with its statistical toolbox version 8.2.

In this study, we examined askers who self-reported anthropometric data while asking a weight-related question, almost exclusively in the “Diet and Fitness” category. Body weight status of askers was classified according to the guidelines for classification of overweight/obesity in adults by the National Institute of Health, defining the normal/healthy BMI range as 18.5 to 25, and partitioning askers into 4 categories: 1) individuals who are overweight (BMI range 25 to 30) as measured by computed BMI (“actually overweight”); 2) persons who are obese (BMI over 30) as measured by computed BMI (“actually obese”); 3) individuals who ask weight-related questions but who are actually normal weight; and 4) persons who are underweight according to computed BMI [32]. If askers were under the age of 18 years, their BMI percentiles were calculated with respect to age and gender based on the Centers for Disease Control and Prevention growth curves [33], [34].

We used term matching to extract questions in which a user asks for community opinion on his/her weight, automatically extracting all questions which contained the phrase: “*Am I* <term>?” with “term” being one of: “*skinny*” (n = 6,189), “*thin”* (n = 17,541), “*fat*” (n = 51,988), and “*obese*” (n = 7,353) in their title or description. Other terms such as “*normal weight*” and “*overweight*” did not return a significant number of questions with anthropomorphic data, and were thus not used in the analyses. As the results show, each weight category is characterized by different average anthropomorphic characteristics. Thus, each is analyzed separately.

We did not attempt to find all questions that may report body measures; instead, we aimed to obtain a highly accurate and focused dataset of similar questions. Written text and content of questions were then automatically scanned to extract the age, gender, weight, and height of the asker. A typical sample question is *“I am a male, 15 years old. I weigh 180 pounds, and am 5 foot 9 inches tall. Am I fat?*” We defined individuals’ “reported weight” as the weight in pounds that askers provided in their posted questions. We defined the “perceived weight” as the inquired weight category of askers, for example, in the following question the “perceived weight” is “skinny”: “*Am I skinny? I am female, 5 foot 4 and weigh 110 pounds?*” Below we refer to the question terms “skinny,” “thin,” “fat,” and “obese” as the perceived/inquired weight category. Respondents who did not report their gender, age, height, and weight or who asked questions in multiple weight categories were excluded, which resulted in the following number of excluded respondents: skinny: 5,965 thin: 16,769, fat: 49,352, obese: 7,059 for a final sample size of 3,926 unique users.

The automatic measurement extractor described above is tuned for precision, rather than recall. This means, for example, that a sentence like “I weigh 128” will not trigger the rule, because the missing suffix “lbs” (or variants thereof). As such, it is more likely to fail to report a measurement rather than report it erroneously. To validate the use of the automatic extractor, 200 randomly selected questions were inspected manually and compared with the results of the automatic extractor. For age, there were 2 errors and 46 omissions. For weight, there was one error and 59 omissions. For height, there were 5 errors (mean error 5.3 inches below actual height) and 27 omissions. There were 6 omissions for males and 16 omissions for females.

We categorized askers by reported weight by labeling askers as having “normal” weight (BMI >18.5 and <25) and other categories using standard CDC thresholds [32]. Seventy-seven percent of inquired self-reported weights were correct by CDC guidelines, a similar accuracy to a recent study which found 75% of respondents correctly classified themselves as overweight/obese [35]. However, the answers dropped in accuracy around a calculated BMI of 18, 25 and 30. We linked county-level data on BMI available via the Centers for Disease Control and Prevention to each asker [36].

We included normal-weight participants who asked whether they are fat or obese as a separate category, as this question may serve as a marker of body image insecurity and can help to differentiate the harmful psychological effects of body image stigma from the physiological effects of overweight/obesity. For example, we examined whether the asker made follow-up inquiries regarding mental health or posed unfounded weight concerns, such as a person with a healthy weight inquiring if he/she is “obese.”

Askers often posted additional questions at a later date, as evidenced by multiple questions posted by the same (obfuscated) user identifier. We stratified askers according to their reported weight (computed BMI) and perceived weight. We then estimated a ratio for the likelihood of posting a question to each future category as the probability of a stratified user posting to a category divided by the same probability for the entire population of askers who asked a weight-related question. These data can be used to gain a better understanding of the asker’s life circumstances. The topics of categories of asker questions that were examined included the following: 1) beauty and style, 2) family and relationships, 3) health, 4) pregnancy and parenting. Given the high rates of stigmatization and bullying reported by obese individuals, we explored whether an asker’s BMI or inquired weight (e.g. “Am I obese?”) predicted additional postings related to bullying [22], [37]. “To examine potential links between weight concerns and bullying, we extracted 43,219 questions that explicitly mentioned bullying in their text (e.g., “How can I make new friends after having been bullied?”). To validate our search for posts about bullying, out of these questions we manually examined a random sample of 300 questions, and identified which of these postings suggested the author was bullied. If the question included statements such as “I'm being bullied”, “Someone is doing X to me. Is this bullying?”, or “I feel I'm being bullied by X”, we marked this as a question where the asker was bullied. Conversely, if the person asked “Is my dog bullying the neighbor's dog”, that was a negative match. In total, 68% of the 300 questions were marked as positive matches”.

Respondents were linked to counties through the zip code they provided to Yahoo upon registration with the website, which was then coded to counties automatically and de-identified. Yahoo Answers user provided zip code data have been proven to be generally accurate in determining users’ location [38]. We examined whether county-level obesity impacted the asker’s accuracy in reporting their inquired weight status. We tested this for each weight classification and separately by gender. Both gender and age were considered to help control for different developmental stages in regards to body image. As additional explanatory variables we used the median per-county income as reported in the 2000 US Census [39], and the percentage of obese people per county as provided by CDC [40]. We note that, although our data are strongly biased towards teenagers and young adults, there is evidence that obesity levels of adults and those of teenagers are correlated [41], [42].

## Results

Table 1 lists the demographic characteristics of the askers. The mean age of the askers was 17.1 years (SD = 5.1) ranging from 13 to 59 years. The mean age for males was 18.4 years (SD = 5.1) and for females was 17.2 years (SD = 5.3). The mean BMI for males was 24.8 (SD = 7.2) and 22.7 (SD = 6.3) for females.

**Table 1:** The analytic sample from Yahoo Answers.

	Inquired (self-perceived) weight, number of askers by gender									
	*Male*				*Female*					
Inquired Weight, “Am I …?”	Skinny	Thin	Fat	Obese	Skinny	Thin	Fat	Obese		
Age Range									Totals	
13–15	38	73	277	54	96	253	812	105	1708	
16–18	22	58	319	26	32	148	351	43	999	
19–25	10	121	271	19	20	87	399	39	966	
25+	2	4	92	4	4	24	113	10	253	
Adult BMI^a^										
<18.5	4	87	3	0	10	23	28	0	155	
18.5–25	4	19	93	2	7	44	236	6	411	
25–30	0	5	107	4	2	10	78	7	213	
30+	0	4	83	12	2	11	72	26	210	
Teenage BMI Percentile^b^										
<5	15	16	24	0	18	46	62	1	182	
5–85	42	83	334	17	95	311	882	52	1816	
85–95	4	17	156	13	11	45	201	37	484	
95+	3	25	159	55	7	22	116	68	455	
									*2937*	

Only askers who provided complete data are represented here. Data are stratified by inquired weight. (N = 3,926).

a Ages 20 and up; adult BMI classifications: [44].

b Ages 13–19; teen BMI classifications: [34], [57].

The median askers’ BMI was in the normal weight range. The average reported weight of askers at a given age was highly correlated with the known average population weight for this age group [43] with an R^2^ of 0.96 (p<.001) for males and 0.88 (p<.001) for females. Similar results were obtained for height (male R^2^ = 0.97, female R^2^ = 0.78, p<.001). This demonstrates that, at least on average, anthropometric information reported by askers is highly accurate and representative of the US population, but their perception of whether they are overweight/obese is not.

**Figure 1:**
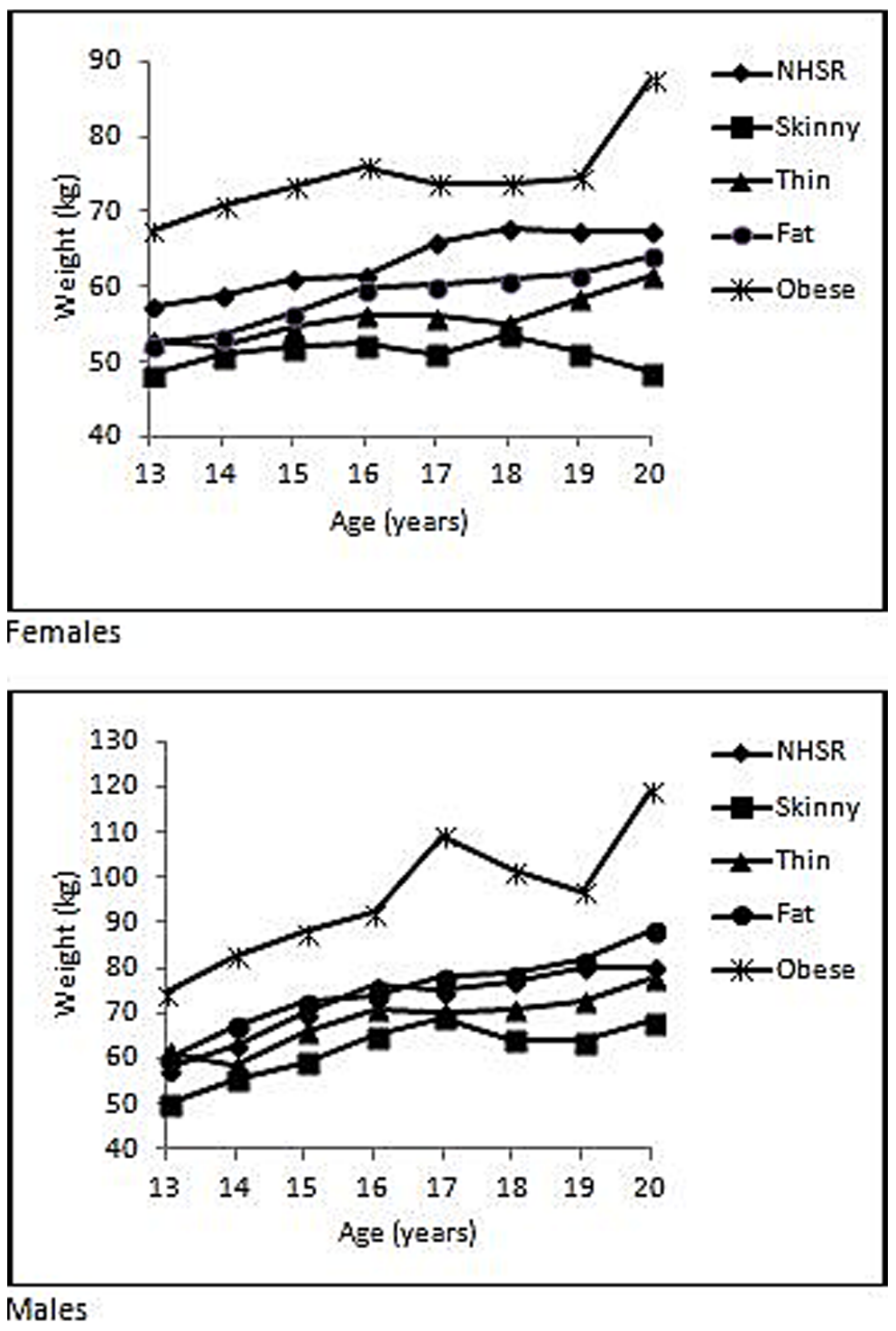
Weight as a function of age by gender, for askers aged 13 to 20, partitioned by perceived weight. NSHR refers to the median weight reported by the CDC [44].

Of men and women who inquired if they were “fat” or “obese,” 68% of men met CDC criteria for overweight/obese, whereas 40% of women met CDC criteria for overweight/obese [44]. Among adolescents, 51% of boys were overweight/obese who asked this question, however, just 30% of girls who asked if they were fat or obese were actually overweight/obese [32]. Thus, compared to men, normal and under-weight women disproportionately questioned if they were, in fact, overweight/obese. This is further exemplified in Figure 1, which shows, by gender, the median weight of askers by age, partitioned by perceived weight. While askers self-perceived as “skinny” and “thin” were below the median weight reported by CDC (2008), and “obese” were above it, males who were self-perceived as “fat” were approximately of equal weight to the median population weight, but females were below it.

### Future Health Postings

Here we report results based on the actual weight/BMI of participants calculated from reported weight and height data, as compared to participants who questioned their weight status, but who were actually normal weight according to established BMI guidelines. Table 2 shows significant differences in postings by gender and actual and perceived weight categories. After dividing the population that posted to weight-related categories into demographic categories, we counted the number of times each subset of this population posted a question to one of the categories on Yahoo Answers. We reported those categories, which received significantly more questions compared to the number of questions asked by the total male and female populations we examined. A topic that received significantly more postings in a specified population was one where the posting rate was statistically significant at p<0.05 False Discovery Rate, a statistical test used in multiple comparison problems to correct for multiple errors [45].

**Table 2:** Significant differences in postings by actual and perceived weight categories.*

	Male				Female			
Actual weight	Underweight	Normal weight		Overweight	Underweight	Normal weight		Overweight
Inquired weight	Underweight	Underweight	Overweight	Overweight	Underweight	Underweight	Overweight	Overweight
**Over-expressed categories**	Parenting	Marriage & divorce	Heart disease	Diabetes	Skin & body			Alternative medicine
		Makeup	Mental health					Trying to conceive
								Pregnancy
								Toddler & preschool
								Newborn & baby
								Wedding
								Parenting

* False Discovery Rate, threshold of 5%.

Women who were actually overweight/obese posted on the broadest range of health issues compared to all men and underweight/normal weight women. Overweight/obese women’s concerns disproportionately focused on the topics of infertility (3.1 times more than the general population of weight-related question askers), parenting (4.1 times more), relationships (4.9 times more), children (3.1 times more), and alternative medicine (2.2 times more). Underweight women disproportionately posted in the “relationships” category (2.0 times more than the general population of weight-related question askers) as well as the “skin and body” category (2.9 times more).

Men posted on relatively few health issues overall. However, overweight/obese men were significantly more likely to post questions about diabetes (4.6 times more than the general population of weight-related question askers). Underweight men were 2.3 times more likely to ask questions about parenting. Normal weight men who asked if they are overweight/obese were disproportionately concerned with mental health (2.1 times more than the general population of weight-related question askers) and heart disease (2.2 times more).

### Bullying

We next explored whether an asker’s BMI predicts questions related to postings on bullying. We extracted an additional set of 43,219 questions which mentioned “bullying” and its variants. These questions were posted by 31,192 distinct askers who mentioned bullying in their text (some askers asked multiple bullying-related questions). This group was older than the average poster, with a mean age of 28 years old. Twice as many females (of any BMI) as males posted questions related to bullying. Those who asked more than one weight-related question were more likely to ask a bullying question than those who asked just one weight-related question. While obese females showed no pattern, men who asked if they were obese were more likely to ask questions about bullying (1.8%, p<0.001, chi^2^), and men who asked if they were overweight were less likely to do so (0.6%, p<0.01, chi^2^) relative to skinny and thin males. The interaction between asking for one’s weight category and gender was statistically significant as a predictor of bullying (p<0.01, chi^2^). Both men and women who were actually overweight/obese were significantly more likely to post on bullying than posters who were normal weight.

**Figure 2:**
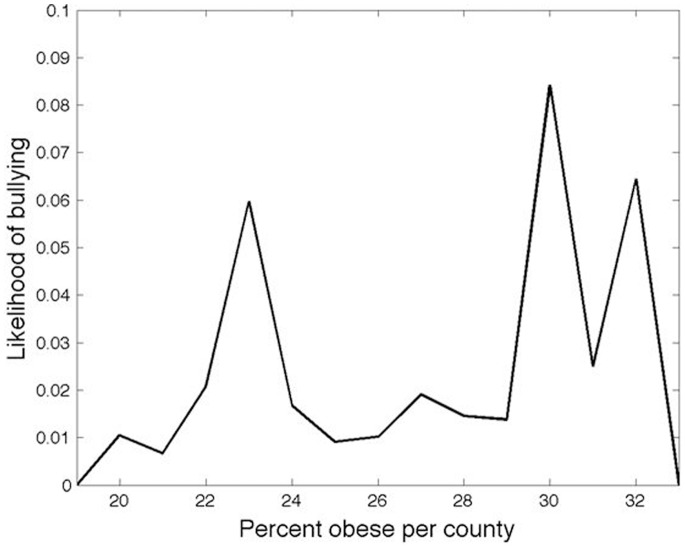
Fraction of askers of weight-related questions who also later asked a bullying-related question, as a function of the percentage of obese people per county [40].

As mean county BMI increased, bullying posts decreased, and then increased again in a bimodal pattern (Figure 2). We attempted to correct the bimodal distribution by transforming county BMI by the absolute difference from the mean across all counties. However, this transformation did not result in a statistically significant variable. Partitioning the distribution shown in Figure 2 by question type, we find that the same bimodal distribution remains for users who asked about low weight categories, but that only the right-handed peak was evident for users who asked about high weight categories. Furthermore, we report that income had a negative relationship with bullying, regardless of BMI, such that the odds of bullying decreased as county-level income increased.

### County-level Effects

Overweight/obese askers who lived in counties with a higher than average proportion of overweight/obese people were more likely to correctly categorize themselves as “fat” or “obese.” However, when partitioned by gender, only overweight/obese females (p<0.01) were significantly more likely to correctly categorize their weight than those located in average or below average weight counties. Normal weight individuals in counties with higher than average levels of overweight/obesity were less likely to ask “am I fat”. These results suggest that higher than average overweight/obesity levels help both normal weight and overweight persons’ to achieve a more accurate body image and more realistic view of one’s weight.

## Discussion and Conclusions

In this paper, we examined whether people who live in counties with higher than average BMIs might experience obesity in different ways than those who are in counties with average or lower than average BMIs. We asked questions surrounding overweight/obesity by examining obesity-related postings in a social Internet media forum, in which respondents post anonymously and on their own volition. This provided the opportunity to explore social attitudes about overweight/obesity–as well as sequelae of public health importance (e.g., bullying)–in a more natural environment than is afforded by scientific surveys.

Overweight/obese women posted significantly more general concerns than the non-obese, and, as would be expected, overweight/obese men expressed significant concerns for obesity-related diseases such as diabetes. Overweight/obese women’s concerns included those linked to obesity such as fertility issues as well as significant interpersonal and sociological concerns regarding relationships suggestive of potential insecurities with image and self as well as broader social stigma.

By some measures, such as wondering whether one is “fat”, living in a county with a higher BMI is protective of one’s health. However, by most measures, it is not. Our study finds that overweight/obese persons exhibit typical “unhealthy” concerns found in previous studies such as apprehension about diabetes and bullying regardless of where they live. However, we do find that normal weight men who perceive themselves to be overweight/obese also express some potentially “unhealthy” obesity-related concerns such as heart disease and mental health issues [5].

Likewise, overweight/obese women were more likely to have future postings on physical or mental health concerns. However, the vast majority of females who inquired if they were “fat” or “obese,” in fact were not. When residing in counties with higher than average levels of overweight/obesity, women’s perceptions of their own overweight/obese status became more accurate compared to counties with lower levels of obesity, a possible sign of better body image. This suggests that people tend to normalize their weight according to others around them, and that overweight/obesity might be less stigmatized in high prevalence areas.

Previous research has demonstrated that when adolescents are surrounded by many overweight peers they may assume their own weight is normal in comparison, and that weight norms and perceptions are influenced by peer-networks [46], [47]. One other study, Ali and colleagues [48], found that people tend to underestimate their actual weight when exposed to higher weight individuals in their county. Ali and colleagues utilized a nationally representative sample. Their data were drawn primarily from Add Health which initially surveyed adolescents in 1994, nearly two decades earlier than our data were captured, and respondents were on average younger and had lower average BMI. The average BMI in our study (24.49) more closely mirrors current national trends in BMI for young people. Likewise, Burke et al., 2010 [49] found a decline in the tendency to self-classify as overweight, despite increases in mean BMI and obesity when comparing the recent wave of the National Health and Nutrition Examination Survey (NHANES) to its predecessor. They suggested that there may be health benefits affiliated with improved body image. We found that overweight/obese persons residing in high BMI counties may also experience benefits, such as reduced stigma, as their body image more accurately represents their actual weight status.

Finally, the more an asker’s body weight deviated from the average BMI (below or above) in a county, the more likely he or she was to subsequently post questions related to bullying. In fact, we found that many young persons who asked weight-related questions of any kind were more likely to ask about bullying. Other work has also shown that lower and higher than average BMI are associated with increased victimization and bullying (see review by Puhl & Heuer, 2009) [20], [22], [23].

In addition to being directed at others in the form of bullying, obesity-associated stigma can also be inwardly directed, possibly leading to physical or mental health problems [50]. Previous work [7] has shown that persons who categorize themselves to be overweight/obese, regardless of their actual weight, may experience negative health outcomes. Our finding that normal weight men who asked whether they are fat or obese disproportionately posted on “heart disease” suggests that worrying about one’s weight may be associated with heart disease and/or unfounded health worries [51].

Our study is exploratory and descriptive and several limitations should be noted. While some research finds BMI to be an appropriate measure of overweight/obesity [52], [53], BMI is a not a perfect measure of obesity, particularly when derived from self-reported data [10], [54]. There is a relatively high correlation between self-reported and measured height and weight [10], [55]. However, women have a tendency to underestimate their weight and men tend to overestimate their height [10], [56]. While we relied on self-reported height and weight, the postings from which these values are derived are anonymous and help-seeking in nature, and may pose better than average accuracy of height and weight. Additionally, comparing users who inquire whether they are underweight/skinny with those of normal weight and those who ask if they are overweight/obese can confound the analysis by comparing a potentially eating disordered population with normal weight and overweight populations.

Although using online data helps to reduce face-to-face bias produced in interviews, the online forum does not allow for collecting direct measures of health functioning such as blood pressure, is only representative of Yahoo Answers askers, and relies exclusively on self-reports [12]. While users of social media are increasingly diverse, our users were predominantly from the United States and were, on average, young adults [17]. Moreover, to measure BMI, we excluded a large number of Yahoo Answers askers because they did not provide gender, age, height, and weight data and this decreased our sample size considerably. Our Yahoo Answers data did not include any data on non-internet users–however, it is noteworthy that mobile-phone web browsing and applications allow users to access social media, including Yahoo without requiring access to a computer [17]. We note that our sample is likely not representative of the US population, for reasons which include access to the Internet, propensity to post questions to the website, etc. However, as we demonstrate, there are excellent correlations between median body measures reported in our data and those obtained through physical measurements of representative populations.

Social media internet databases reflect an under-utilized outlet by researchers studying obesity. They provide unique opportunities to examine the public mind-set about body weight and related health and social issues. Our findings suggest that many individuals post obesity-related questions, and these questions are particularly common among young adults. In light of our findings on bullying and obesity-associated stigma, there may be a need for public health messages and policies that encourage not only healthy body weight, but healthy body image, as well as interventions to reduce weight-related bullying.

Counties with higher-than-average BMI resulted in the least proportion of persons questioning their weight and more accuracy among those who did ask about their weight. As such, normal and below average BMI counties, who had the lowest percentage of persons correctly classifying their weight, may especially benefit from supportive public health messages.

As social media continues to become a central forum for social interaction and knowledge exchange, researchers may be better able to monitor and address obesity-related stigma. The internet serves as a powerful source of information for understanding the impact of obesity on society, not just by measuring people’s waistlines, but also the psychological threats posed to obese people by both the general public and health professionals alike.
